# First Complete Genome Sequence of Methicillin-Resistant *Staphylococcus aureus* Strain SO-1977 Isolated from Khartoum, Sudan

**DOI:** 10.1128/genomeA.00945-17

**Published:** 2017-10-19

**Authors:** Sofia B. Mohamed, Mohamed S. Ali, Faisal M. Alamir, Tahani B. Alyas, Abdallah E. Ahmed, Almeen O. Seed, Rihab A. Omer

**Affiliations:** a National University Research Institute-National University, Khartoum, Sudan; b Tropical Medicine Research Institute, Khartoum, Sudan

## Abstract

Methicillin-resistant *Staphylococcus aureus* is increasingly becoming resistant to most antibiotics and consequently has become a challenging public health problem in Sudan. The present study documented the first complete genome sequence of strain SO-1977, isolated from a contaminated wound in Sudan.

## GENOME ANNOUNCEMENT

Methicillin-resistant *Staphylococcus aureus* (MRSA) is a worldwide spreading pathogen. It is considered the main communal and hospital-acquired cause of morbidity and mortality. Globally, the spread of epidemic MRSA has become a clinical concern, leading to a number of community-associated problems (1). In Sudan, MRSA infection is a hospital-associated epidemic with an increasing incidence rate (2). Weaknesses in available typing methods used to identify modes of transmission and control of MRSA have impeded effective infection prevention and control. However, the high resolution offered by whole-genome sequencing has the potential to improve the understanding and management of MRSA infections (3). A sample of MRSA was collected from a wound in a 25-year-old patient in a hospital in Sudan. The DNA was sequenced by Illumina MiSeq with a genomic library of 929-bp inserts. Genome assembly by SPAdes (4) and ARAGORN was performed for predicting tRNA (5). RNAmmer (6), BLAST (7), and HMMER (8) were subsequently used to predict rRNA and protein-coding genes. The 16S rRNA sequence in the SO-1977 strain was compared with 16S rRNA sequences of different strains in NCBI. A total of 885,995 quality reads resulted in 122.26-fold overall coverage. The genome was 2,827,644 bp with a 32.8% GC content. The number of predicted coding sequences (CDS), tRNAs, and rRNAs were 2,629, 51, and 4, respectively. The 16S rRNA of the SO-1977 strain shared 99.9% identity with the JH1, N315, S33R, and ATCC 12600 strains, and 100% identity with NBRC 100910. This is the first complete genome sequence of MRSA strain SO-1977 isolated from a human wound in Sudan. Advanced analyses of this genome may improve our understanding of molecular mechanisms such as antibiotic resistance and pathogenicity.

### Accession number(s).

This whole-genome shotgun project has been deposited at DDBJ/ENA/GenBank under the accession number NFZY00000000. The version described in this paper is version NFZY01000000.
